# Processes Underlying Rabies Virus Incursions across US–Canada Border as Revealed by Whole-Genome Phylogeography 

**DOI:** 10.3201/eid2309.170325

**Published:** 2017-09

**Authors:** Hannah Trewby, Susan A. Nadin-Davis, Leslie A. Real, Roman Biek

**Affiliations:** Emory University, Atlanta, Georgia, USA (H. Trewby, L.A. Real); University of Glasgow, Glasgow, Scotland, UK (H. Trewby, R. Biek);; Canadian Food Inspection Agency, Ottawa, Ontario, Canada (S.A. Nadin-Davis)

**Keywords:** raccoon rabies virus, rabies, phylogeography, transboundary outbreaks, whole-genome sequencing, wildlife disease, spatial epidemiology, viruses, zoonoses, Canada, United States

## Abstract

Disease control programs aim to constrain and reduce the spread of infection. Human disease interventions such as wildlife vaccination play a major role in determining the limits of a pathogen’s spatial distribution. Over the past few decades, a raccoon-specific variant of rabies virus (RRV) has invaded large areas of eastern North America. Although expansion into Canada has been largely prevented through vaccination along the US border, several outbreaks have occurred in Canada. Applying phylogeographic approaches to 289 RRV whole-genome sequences derived from isolates collected in Canada and adjacent US states, we examined the processes underlying these outbreaks. RRV incursions were attributable predominantly to systematic virus leakage of local strains across areas along the border where vaccination has been conducted but also to single stochastic events such as long-distance translocations. These results demonstrate the utility of phylogeographic analysis of pathogen genomes for understanding transboundary outbreaks.

Control measures are often used at the edges of a pathogen’s range to limit geographic spread and prevent incursions of infection into areas free from disease. Although geopolitical boundaries generally do not directly affect spread of infectious diseases, human-imposed control measures are often structured around national or international borders. Where such control measures fail, the resulting outbreaks can prove extremely costly in terms of public health and economic and political consequences. It is therefore vital to understand the events involved in such transboundary outbreaks of infection and particularly how these events relate to the control measures applied at the boundary.

Rabies virus is a major zoonotic pathogen worldwide. In the United States, the geographic range of the raccoon variant of rabies virus (RRV) has expanded in recent decades and is now endemic throughout the eastern seaboard area (*1*). Further spread of RRV has been largely contained through oral vaccination of raccoons along the edge of its range (*2*). However, multiple incursions across the vaccination corridor have occurred at the northern edge of the RRV range, corresponding to the US–Canada border; the resulting outbreaks in the Canadian provinces of Ontario, Quebec, and New Brunswick have necessitated large-scale control operations to prevent the establishment of RRV in Canada. By focusing specifically on these outbreaks of RRV in Canada, we are by default highlighting points at which transboundary controls have failed. However, our doing so does not imply that the control measures at the Canada border have been unsuccessful. Despite the epidemic expansion of RRV covering 40–60 km/year in the absence of controls (*3*–*6*), spatial spread of RRV has been static in most areas of the Canada border for >15 years (*7*).

RRV was first reported in the US state of New York in 1990. By 1994, it had spread to reach the Canada border at Niagara in western New York, and by 1996, it had reached the New York–Ontario border at the St. Lawrence River. Implementation of rabies vaccination started along the Niagara River in 1994 and at potential crossing points along the St. Lawrence River from 1995 on, later replaced by larger scale oral vaccination (*8*). The first RRV outbreak in Ontario occurred in the southeastern part of the province during 1999–2005; 126 cases were confined to an area of the mainland adjacent to the St. Lawrence River, and 6 cases occurred on Wolfe Island between Ontario and New York at the mouth of Lake Ontario (*8*,*9*). More recently, in 2015, an outbreak was identified west of the Niagara area and is ongoing (*7*).

At the US–Canada border between Quebec and Vermont, oral rabies vaccination was implemented in the late 1990s in response to the northward spread of RRV through Vermont. The first outbreak of RRV in Quebec occurred during 2006–2009, near the Vermont border (*10*). Another isolated case of raccoon rabies was reported in 2015 at the border with New York in southwestern Quebec (*7*).

In New Brunswick, RRV outbreaks occurred during 2000–2002 and 2014–2016, both in the southwestern part of the province near the border with Maine (*7*). RRV vaccination was conducted at the New Brunswick–Maine border from 2001 through 2008 and is currently in place after the 2015 outbreak.

In this study, we used RRV whole-genome sequences to investigate the processes giving rise to these outbreaks in Canada, particularly with respect to the effectiveness of the vaccine area at the US–Canada border, and to determine whether these processes were comparable across different outbreaks. We generated 289 RRV whole-genome sequences: 140 sequences from RRV cases covering each of the Canada outbreaks and 149 sequences from cases in the neighboring US states of New York, Vermont, and Maine. Using Bayesian phylogeographic approaches, we addressed the following 3 questions: 1) Did the Canada outbreaks result from multiple simultaneous incursions of RRV or from single introductions? 2) Is there evidence of backflow of RRV from Canada into the United States? 3) Did the Canada outbreaks originate from RRV lineages circulating locally, or are they attributable to long-distance movement?

## Materials and Methods

### Samples and Sequencing

In eastern Canada, brain samples from animals suspected to be infected with rabies virus are submitted to the Centre of Expertise for Rabies of the Canadian Food Inspection Agency in Ottawa for diagnosis. In addition, any rabies-positive wildlife cases diagnosed by provincial authorities during enhanced surveillance activities are confirmed by the laboratory in Ottawa. We selected a temporally and spatially representative subset of RRV samples for sequencing from the Canada outbreaks in Ontario (n = 57), Quebec (n = 51), and New Brunswick (n = 32). For comparison, we obtained another set of confirmed rabies virus samples, in most cases collected over the same period as the Canada outbreaks, for sequencing from the relevant state rabies laboratories of New York, Vermont, and Maine. The high-density sampling in New York and Vermont enabled us to focus sampling on cases within 75 km of the Canada border in these 2 states (54 sequenced samples in New York and 62 in Vermont), with the aim of capturing representatives of most RRV lineages circulating near the border. A lower density of samples was available from Maine, collected in 2013 and 2014 only, and therefore we sequenced RRV from throughout this state (33 sequences). Details of sequenced samples are shown in Technical Appendix Table 1.

We performed RRV genome extraction and sequencing as described in detail by Nadin-Davis et al. (*11*). Viral RNA was extracted from brain tissue of animals with confirmed infection by using Trizol (Life Technologies Inc., Carlsbad, CA, USA) and further purified by using a MagMax instrument (Applied Biosystems, Foster City, CA, USA). RNA was amplified as 3 overlapping amplicons covering the whole RRV genome. Purified amplicons from a single sample were pooled and used to generate indexed libraries with Nextera XT kits (Illumina, Inc., San Diego, CA, USA); libraries were sequenced as 200- or 250-bp paired end reads on an Illumina MiSeq machine. Genomes were sequenced with high depth of coverage (average >1,000×), and reference-based assembly was conducted by use of the NGen program in the DNASTAR Lasergene software package, version 11 (*12*), with either the RRV reference genome (GenBank accession no. EU311738) or more genetically related sequences generated during this study that better facilitated complete assembly, to generate consensus sequences.

### Phylogeographic Analyses

To estimate RRV transitions between geographic regions, we conducted discrete trait phylogeographic analysis (*13*). Sequences were grouped into 8 groups according to location: western Ontario, eastern Ontario, western New York, northwestern New York, New Brunswick, Maine, Quebec, and Vermont (this latter group includes 2 New York sequences, New York.1995.3745 and New York.2011.5590 [Technical Appendix Table 1], which clustered geographically and genetically with Vermont sequences). Independent incursions into Canada were identified as lineages of the maximum clade credibility phylogeny stemming from a most recent common ancestor with a >90% posterior probability of occurring in Ontario, Quebec, or New Brunswick, according to discrete trait ancestral state reconstruction.

We conducted analyses in BEAST version 1.8.2 (*14*) with the BEAGLE library (*15*) by using the generalized time-reversible model with gamma distributed rate variation among sites and separate partitions for coding and noncoding regions and a relaxed molecular clock (*12*) with branch rates drawn from an exponential distribution (identified by model selection as the best fitting models for these data; Technical Appendix text and Table 2). Asymmetric transition rates were allowed between regions. Significant transitions were identified by using Bayesian stochastic search variable selection to calculate Bayes factors in the program SPREAD version 1.0.6 (*16*), and the number of transitions between regions was estimated by using Markov Jump counts (*17*).

### Identifying Long-Distance Movement

If an outbreak in Canada were the result of local spread of infection, we would expect the responsible viruses to be genetically similar to US RRV lineages circulating near the Canada border. However, even where dense sampling and sequencing was conducted near the Canada border (i.e., in New York and Vermont), sampling was not exhaustive. Therefore, the absence of a virus closely related to that causing the Canada outbreak in neighboring US regions may indicate an outbreak initiated by long-distance virus movement but could also be the result of incomplete sampling of RRV transmission chains circulating in the local area. We extracted the coalescent time between the most recent common ancestor of a Canada outbreak and the most genetically similar US sequence as a measure of the length of time the outbreak lineage had been circulating unsampled. To generate an expectation for the length of time lineages would circulate undetected under our sampling regimen, we took the distribution of coalescent times for US sequences within each of the 3 major clades. Coalescent times falling outside the 95th percentile interval of US times indicate an outbreak that was probably initiated by long-distance movement. Depending on the (unknown) level of long-distance movement underlying the US samples, this 95th percentile might be overly conservative in identifying long-distance movements.

## Results

**Figure 1:**
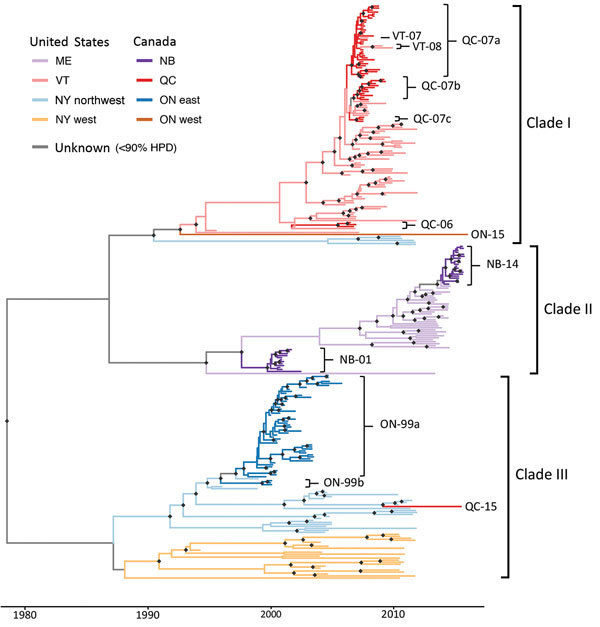
Time-scaled maximum clade credibility phylogeny of sequenced genomes of raccoon-specific variant of rabies virus, US–Canada border. Branches are colored by inferred geographic region. Samples belonging to Canada lineages are labeled by province and year of first sample, as is backflow of infection from Canada into Vermont. Black diamonds indicate nodes with >90% posterior support. HPD, highest posterior density; NB, New Brunswick; ON, Ontario; QC, Quebec.

The sequenced samples fell into 3 well-supported major clades (Figure 1) that are largely structured by region. Clade I consists predominantly of sequences from Quebec and Vermont, although it also includes 5 sequences from northwestern New York and from the 1 case in western Ontario (Ontario-15); clade II is restricted to samples from New Brunswick and Maine; and clade III contains samples from Ontario and New York, with the addition of the 1 isolate from western Quebec in 2015 (Quebec-15). Clades I and III correspond to lineages identified in a previous study of RRV in New York (*18*); clade I corresponds to lineage 3A (found in southeastern New York in the previous study) and clade III to 3B (found in western and northern New York). Because our sampling scheme focuses on RRV infections near the Canada border in western and northern New York, only 5 of the samples from New York sequenced here fall into clade I.

BEAST analysis revealed a molecular clock rate of 3.28 × 10^−4^ nucleotide substitutions/site across the genome for these sequences (95% highest posterior density [HPD] 2.83 × 10^−4^ to 3.76 × 10^−4^). The time to most recent common ancestor was estimated as 1990 for clade I (95% HPD 1983–1994), 1994 for clade II (95% HPD 1988–1998), and 1987 for clade III (95% HPD 1979–1991). These estimates suggest that the 3 clades were probably already diverged before RRV entered the region, as indicated by Szanto et al. (*18*).

We identified 10 independent incursions of RRV, giving rise to the 6 Canada outbreaks, from the maximum clade credibility tree (Figure 1). We analyzed each of these outbreaks to determine the number of recorded cases and whether evidence exists for multiple virus introductions, backflow to the United States, or long-distance movement (Table).

**Table:** Summary of Canada outbreaks and evidence of multiple introductions of raccoon-specific variant of rabies virus, backflow from Canada to the United States, and long-distance movement initiating an outbreak

Location	Time	No. recorded cases	Evidence of multiple introductions	Evidence of backflow to the United States	Evidence of long- distance movement
Ontario (east)	1999–2005	132	Yes	No	No
Ontario (west)	2015–ongoing	307*	No	No	Yes
Quebec	2006–2009	104	Yes	Yes (strong)	No
Quebec	2015–2015	1	No	No	No
New Brunswick	2000–2002	64	No	No	No
New Brunswick	2014–ongoing	30*	No	Yes (weak)	No

*Number correct as of April 30, 2017.

### Ontario

**Figure 2:**
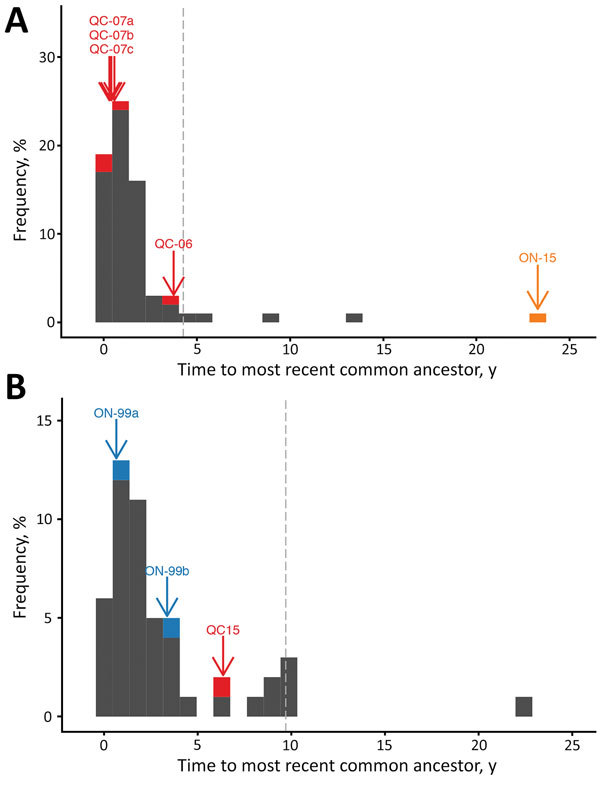
Distribution of coalescent times for raccoon-specific variant of rabies virus near the US–Canada border, clade I (A) and clade III (B). Gray histograms give the distribution of coalescent times for each US sample in the clade, and colored bars and labels indicate the coalescent times for the most recent common ancestor of each Canada lineage in the specified clade. Gray dashed lines indicate the 95th percentiles of the coalescent times for virus from the United States. ON, Ontario; QC, Quebec.

Ontario was subject to a persistent outbreak of RRV from 1999 through 2005, resulting in reported cases on Wolfe Island and the Leeds/Grenville area in mainland Ontario. Discrete traits reconstruction (Figure 1, eastern Ontario outbreak shown in dark blue) suggested that the Wolfe Island viruses (Ontario-99b) are part of a separate incursion to the mainland (Ontario-99a), confirming previous suggestions from partial genome data (*19*). Our results give no indication that the mainland Ontario incursion was caused by multiple invading lineages, and we found no evidence for backflow of infection from Ontario to the United States (online Technical Appendix Figure 1). Sequences from the eastern Ontario outbreak were genetically closely related to sequences circulating locally on the US side of the border with eastern Ontario (Figure 2), suggesting that local spread of infection is responsible for initiating this outbreak. The spatial-genetic spread of this Ontario outbreak is described in more detail by Nadin-Davis et al. (*11*).

By contrast, the outbreak in western Ontario (represented here by 1 sequence, Ontario-15) falls into a completely separate clade (clade I) than other viruses circulating in the neighboring area of New York (clade III; light orange branches in Figure 1). Even in comparison with other sequences in clade I, the Ontario-15 sequence is considerably divergent; coalescent time is >20 years, and it falls distinctly outside the 95th percentile of coalescent times for US sequences in this clade (Figure 2). These findings provide evidence that the variant represented by isolate Ontario-15 was the result of long-distance movement from outside the study area, rather than local spread across the border. Although our results provide some statistical support for the Ontario-15 incursion originating in Vermont (online Technical Appendix Figure 1), a more full exploration of the origins of the Ontario-15 incursion would require a more comprehensive study in which sampling is not restricted to RRV samples within 75 km of the Canada border. An additional isolate from this ongoing outbreak, sequenced subsequent to preparation of this article, differs from the Ontario-15 isolate at 13 of 11,924 sites. This finding represents a genetic similarity of 99.9%, which would place these 2 samples in the same phylogenetic group (data not shown).

### Quebec

**Figure 3:**
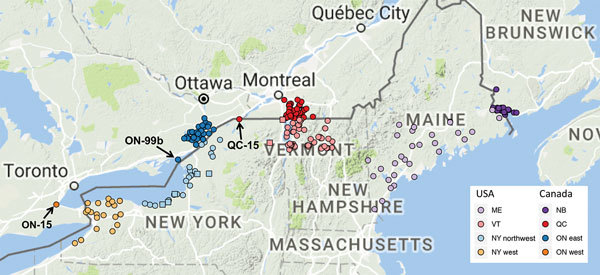
Locations of sequenced samples from Canada outbreaks of raccoon-specific variant of rabies virus infection in western Ontario (n = 1), eastern Ontario (n = 56), Quebec (n = 51), and New Brunswick (n = 32); and from the United States within 75 km of the border in western New York (n = 23), northwestern New York (n = 29, including 5 samples into clade I, indicated by squares), and Vermont (n = 64, including 2 samples from New York that grouped within this clade, indicated by squares); and from throughout Maine (n = 33). Map generated by using ggmap package (*20*). NB, New Brunswick; ON, Ontario; QC, Quebec.

With the exception of the 1 case in eastern Quebec in 2015 (Quebec-15), all cases of RRV infection from Quebec were reported from 2006 through 2009 and were again focused on a relatively small area (Figure 3). The combination of the high level of discrimination provided by whole-genome sequencing plus high-density sequencing of samples from close to the Canada border in this study have made it possible to attribute this single temporal outbreak to several separate incursions of RRV into Quebec (lineages Quebec-06, Quebec-07a, Quebec-07b, and Quebec-07c; Figure 1). The last 2 reported cases of RRV in this outbreak, although located near the border, are shown here to be part of an ongoing circulation of lineage Quebec-07b within Quebec, rather than a separate introduction, as might have been assumed in the absence of sequencing.

Incursions of lineages Quebec-07a, Quebec-07b, and Quebec-07c involved viruses closely genetically related to others circulating locally near the Quebec border (Figure 2), indicating that these were probably the result of local spread from RRV circulating near the border. Although the Quebec-06 lineage seems more genetically distinct from other sequences circulating near the border (and also from the other Quebec lineages; Figure 1), it does fall within the 95th percentile interval for coalescent times in this clade (Figure 2, panel A); therefore, there is no evidence to rule out the possibility that local spread from Vermont initiated this incursion. Phylogenetic analysis using discrete traits provides strong statistical support (Technical Appendix Figure 1) for backflow of RRV infection from the Quebec outbreak back into the United States. These instances of backflow (Vermont-07 and Vermont-08; Figure 1) were each identified within 30 km of the Quebec border, again consistent with local transmission of disease.

The 1 case reported in 2015, Quebec-15, was located at the western edge of the Quebec border with the United States. Contrary to the previous Quebec outbreak, the sequence for this case falls into clade III (Figure 1), indicating that it is linked to the lineage that has spread north through New York, as opposed to the predominantly Vermont-associated clade I implicated in the 2006–2009 outbreak. This finding is not surprising because Quebec-15 was found in an area directly adjacent to New York (Figure 3) and was reported soon after cases of RRV reached Franklin County, New York, adjacent to the Quebec border. The Quebec-15 case was located in an area where vaccination had previously not been conducted and has not been followed by any further reports of RRV in Quebec. Although on first examination the Quebec-15 sequence does exhibit some divergence from other sequences in the clade, it is again within the 95th percentile of coalescent times for this clade (Figure 2, panel B), further confirming that this case is probably the result of local spread of viruses from the New York side of the Quebec border.

### New Brunswick

Our results provide no evidence that either of the outbreaks in New Brunswick (2000–2002 and 2014–2015) was initiated by multiple incursions into New Brunswick; however, discrete trait phylogeographic analysis gives some limited statistical support for backflow of RRV from New Brunswick into Maine (Technical Appendix Figure 1; note that this backflow is not represented in the maximum clade credibility tree in Figure 1). Sequences from the second New Brunswick outbreak seem closely genetically related to sequences circulating in Maine; however, to examine further the suggestion of backflow from New Brunswick into Maine, and to assess whether the 2000–2002 outbreak is the result of local spread or of long-distance translocation of infection as was suggested at the time (*21*), more extensive sampling and sequencing of RRV from cases near the New Brunswick border would be necessary. 

## Discussion

The use of high-throughput RRV sequencing enabled us to investigate events giving rise to a series of transboundary RRV outbreaks in eastern provinces of Canada that border the United States. By generating high-resolution whole-genome sequences and comparing results across multiple Canada outbreaks, we were able to discriminate between different epidemiologic scenarios and gain generalizable insights that would not be possible to gain from single-outbreak data.

In most instances, results were consistent with the outbreaks being initiated by 1 lineage, possibly representing 1 infected individual. A major exception was the outbreak in Quebec in 2006–2009, which involved at least 4 cross-border incursions. The Quebec 2006–2009 outbreak also showed strong evidence of backflow of infection from Quebec across the border into the United States. The combination of multiple introductions and backflow of infection indicates that the Quebec–Vermont border and adjacent areas of vaccination were relatively permeable to the spread of RRV during 2006–2008. These results suggest that this particular outbreak was related to systematic challenges in maintaining an effective vaccine corridor at this location and time, as opposed to rare stochastic events. A likely contributing factor for the higher transboundary transmission in Quebec is the lack of natural barriers along this border, compared with the major rivers or lakes that reinforce the border between Ontario and the United States. A similar argument can be made for the 1999–2005 Ontario outbreak, given the evidence for 2 separate introductions into Wolfe Island and the mainland, although no indications of backflow or further introductions were found. Weak evidence for backflow of RRV into the United States was also found for New Brunswick, although more in-depth sampling from Maine would be necessary to confirm this; other outbreaks showed no indication of backflow of infection into the United States. Transmission of infection in both directions across the US–Canada border highlights the need for coordination of surveillance programs. For future detection of such backflow events, however, surveillance strategies will probably need detailed genomic data and dense geographic sampling, as described here for New York and Vermont.

Introductions of RRV into Canada were predominantly attributable to viruses closely genetically related to lineages circulating near the US–Canada border. This finding indicates that for many outbreaks, whether multiple introductions or backflow of RRV were evident in the data, the largest risk for introduction stems from local pressure of infection resulting in RRV spreading through the areas of vaccination across the international boundary. This finding is consistent with previous findings of an observed breach of the vaccine corridor within the United States into the state of Ohio, which also implicated local spread of virus lineages through the vaccinated area (*22*). However, we demonstrate that at least 1 introduction into Canada (Ontario-15) was attributable to movement of infection across an exceptionally large distance. The index case was found 64 km from the Ontario–US border, and our results indicate that it is highly unlikely to have originated from US territory within the scale of our sampling (75 km from the border). Most raccoon movements are <5 km (*23*–*27*); however, long-distance dispersal of raccoons covering >100 km has been reported and is generally attributed to human-mediated translocation, whether deliberate or inadvertent (*4*,*28*,*29*). The Ontario-15 introduction was adjacent to the Niagara region, an area where vaccination has been conducted on both sides of the border for over a decade and where the border is further strengthened by the large Niagara River. It is also the area on the US–Canada border that was first reached by the northward expansion of RRV in 1994, resulting in the longest potential for transboundary incursions. The absence of any local spread of infection suggests that the local barrier to transboundary incursion of RRV here is particularly strong. However, our results highlight that such areas are still vulnerable to long-distance translocation events, effectively allowing RRV infection to bypass areas of vaccination completely.

Long-distance translocations are likely to be stochastic events and therefore difficult to predict and prevent; however, on the basis of our evidence, these events seem to be relatively rare compared with breaches of the vaccination corridor by locally circulating viruses. Although such breaches could occur anywhere along the US–Canada border, it is apparent that some areas experience multiple incursions, either in short succession (Ontario, Quebec) or separated by several years (New Brunswick), suggesting deterministic factors. Identification of these factors, which are probably related to temporal and spatial variation in raccoon demography or vaccination coverage affecting local pressure of infection, is an area for future work. 

Irrespective of the underlying mechanisms, these results demonstrate the utility of whole-genome data and bioinformatics approaches for resolving transmission processes in sensitive areas such as international borders for infectious diseases of high public concern. Increased efforts are needed to make these tools available to government agencies dealing with transboundary diseases and to facilitate international collaboration toward controlling and ultimately eliminating the spread of infection.

Technical Appendix Supplementary information for phylogeographic analysis of virus genomes and processes underlying rabies virus incursions across US–Canada border.
