# Supplementary material for: Processes Underlying Rabies Virus Incursions across US–Canada Border as Revealed by Whole-Genome Phylogeography
Source: Emerg Infect Dis. 2017 Sep;23(9):1454–61. doi: 10.3201/eid2309.170325 (PMC5572885; doi:10.3201/eid2309.170325)
Supplement: Technical Appendix — Supplementary information for phylogeographic analysis of virus genomes and processes underlying rabies virus incursions across US–Canada border. [file 17-0325-Techapp-s1.pdf]

Article DOI: https://doi.org/10.3201/eid2309.170325

Processes Underlying Rabies Virus Incursions across US–Canada Border as Revealed by Whole-Genome Phylogeography

Technical Appendix

Supporting Information

Phylogenetic model selection

The appropriate nucleotide substitution model for these data was identified using jModelTest v2.1.7 (*1*). The GTR model with gamma-distributed rate variation among sites (GTR+G) was identified as the best fitting nucleotide substitution model by both BIC and DIC methods, and as the second best fitting model by AIC and AICc methods (Δ AIC <2.5). A maximum likelihood (ML) phylogeny was then generated in PhyML v3.0 (*2*) using the GTR+G model of nucleotide substitution. The R^2 correlation between root-to-tip distance and date of sampling in the resulting phylogeny was 18.7%, indicating a measurably evolving signal within these data.

Further model selection was carried out in BEAST v1.8.2 (*3*) to identify the most appropriate gene and codon partitions, and the best fitting molecular clock model for the data. The following gene and codon partitions were tested, with variation in substitution rate, rate heterogeneity, and base frequencies allowed between partitions:

1) Partitions for concatenated non-coding regions and for each for the five genes separately, with:

   a) Three separate partitions for codon positions (CPs) 1, 2 and 3 separately

   b) No codon partitions

2) Separate partitions for concatenated non-coding regions and concatenated gene regions with:

   a) Three separate partitions for codon positions (CPs) 1, 2 and 3 separately

b) No codon partitions

3) No gene or codon partitions.

Each of these sets of gene/codon partitions was tested with a relaxed molecular clock with branch rates drawn from an exponential distribution (UCED clock); and a relaxed molecular clock with branch rates drawn from a log normal distribution (UCLD clock) (*4*). Tip dates were calibrated using the date associated with each sample in the laboratory records.

Each model was run in duplicate using the GTR+G nucleotide substitution model and the ML phylogeny (above) as starting tree, and the Bayesian skyline model as a flexible demographic prior (*5*). The clock rate prior was set to a normal distribution with mean of 1.44×10^{-4} nucleotide substitutions per site (based on results from [*6*]), truncated to 0 and 0.15 nucleotide substitutions per site, and with a wide standard deviation of 0.0144 to allow for variation from the prior mean.

BEAST analyses were run until MCMC chains had converged, as determined by visual checking in Tracer v1.6.0 and by effective sample size values greater than 200. Model selection was carried out using marginal likelihood estimates as generated by Path Sampling and Stepping Stone Sampling (*7*,*8*).

The model selection analysis for the UCLD clock with five gene partitions and three separate partitions for each codon position (1b) failed to converge. Results for the other models tested (Table S2) indicate that the UCED molecular clock, with separate partitions for coding and non-coding regions, but no partitioning by codon position (2b) was best fitting model for these data.

References

1. Darriba D, Taboada GL, Doallo R, Posada D. jModelTest 2: more models, new heuristics and parallel computing. [Nature Publishing Group.]. Nat Methods. 2012;9:772. PubMedhttp://dx.doi.org/10.1038/nmeth.2109

2. Guindon S, Gascuel O. A simple, fast, and accurate algorithm to estimate large phylogenies by maximum likelihood. Syst Biol. 2003;52:696–704. PubMedhttp://dx.doi.org/10.1080/10635150390235520

3. Drummond AJ, Suchard MA, Xie D, Rambaut A. Bayesian phylogenetics with BEAUti and the BEAST 1.7. Mol Biol Evol. 2012;29:1969–73. PubMedhttp://dx.doi.org/10.1093/molbev/mss075

4. Drummond AJ, Ho SYW, Phillips MJ, Rambaut A. Relaxed phylogenetics and dating with confidence. PLoS Biol. 2006;4:e88. PubMedhttp://dx.doi.org/10.1371/journal.pbio.0040088

5. Drummond AJ, Rambaut A, Shapiro B, Pybus OG. Bayesian coalescent inference of past population dynamics from molecular sequences. Mol Biol Evol. 2005;22:1185–92. PubMedhttp://dx.doi.org/10.1093/molbev/msi103

6. Brunker K, Hampson K, Horton DL, Biek R. Integrating the landscape epidemiology and genetics of RNA viruses: rabies in domestic dogs as a model. Parasitology. 2012;139:1899–913. PubMedhttp://dx.doi.org/10.1017/S003118201200090X

7. Baele G, Lemey P, Bedford T, Rambaut A, Suchard MA, Alekseyenko AV. Improving the accuracy of demographic and molecular clock model comparison while accommodating phylogenetic uncertainty. Mol Biol Evol. 2012;29:2157–67. PubMedhttp://dx.doi.org/10.1093/molbev/mss084

8. Baele G, Li WLS, Drummond AJ, Suchard MA, Lemey P. Accurate model selection of relaxed molecular clocks in bayesian phylogenetics. Mol Biol Evol. 2013;30:239–43. PubMedhttp://dx.doi.org/10.1093/molbev/mss243

Technical Appendix Table 1. Detailed information for sequenced samples

Sequence ID	Accession no.	Date of testing	Host species	Country	Province/ State	County	Town	Geographic group
ME.2013.0002		2013-01-03	Skunk	USA	Maine	Kennebec	China	ME
ME.2013.0025		2013-02-04	Raccoon	USA	Maine	York	Wells	ME
ME.2013.0034		2013-02-13	Raccoon	USA	Maine	Penobscot	Dixmont	ME
ME.2013.0052		2013-02-28	Skunk	USA	Maine	Penobscot	Milford	ME
ME.2013.0064		2013-03-21	Raccoon	USA	Maine	Kennebec	Litchfield	ME
ME.2013.0067		2013-03-22	Raccoon	USA	Maine	Somerset	Skowhegan	ME
ME.2013.0098		2013-04-12	Skunk	USA	Maine	York	Eliot	ME
ME.2013.0101		2013-04-17	Skunk	USA	Maine	Cumberland	Windham	ME
ME.2013.0102		2013-04-17	Raccoon	USA	Maine	Hancock	Mariaville	ME
ME.2013.0109		2013-04-25	Raccoon	USA	Maine	Cumberland	Windham	ME
ME.2013.0110		2013-04-26	Raccoon	USA	Maine	Androscoggin	Lewistion	ME
ME.2013.0113		2013-04-30	Skunk	USA	Maine	Franklin	Farmington	ME
ME.2013.0131	KY026414	2013-05-21	Raccoon	USA	Maine	Waldo	Northport	ME
ME.2013.0157		2013-06-06	Red Fox	USA	Maine	Oxford	Hiram	ME
ME.2013.0158		2013-06-10	Skunk	USA	Maine	Waldo	Waldo	ME
ME.2013.0187		2013-06-25	Raccoon	USA	Maine	Lincoln	Southport Island	ME
ME.2013.0233		2013-07-19	Raccoon	USA	Maine	Oxford	Stow	ME
ME.2013.0247		2013-07-31	Gray Fox	USA	Maine	Cumberland	South Portland	ME
ME.2013.0332		2013-08-19	Raccoon	USA	Maine	Kennebec	Monmouth	ME
ME.2013.0425		2013-10-11	Skunk	USA	Maine	Hancock	Franklin	ME
ME.2013.0444		2013-10-28	Skunk	USA	Maine	Franklin	New Portland	ME
ME.2013.0491		2013-12-20	Skunk	USA	Maine	Kennebec	Sidney	ME
ME.2013.0498		2013-12-31	Skunk	USA	Maine	Cumberland	Scarborough	ME
ME.2014.0038		2014-02-27	Raccoon	USA	Maine	Cumberland	North Yarmouth	ME
ME.2014.0079		2014-04-22	Skunk	USA	Maine	Penobscot	Old Town	ME

Sequence ID	Accession no.	Date of testing	Host species	Country	Province/ State	County	Town	Geographic group
ME.2014.0084		2014-05-02	Raccoon	USA	Maine	Somerset	Cornville	ME
ME.2014.0090		2014-05-07	Skunk	USA	Maine	Somerset	Cornville	ME
ME.2014.0091		2014-05-08	Raccoon	USA	Maine	Washington	Charlotte	ME
ME.2014.0095		2014-05-13	Raccoon	USA	Maine	Kennebec	Sidney	ME
ME.2014.0103		2014-05-22	Raccoon	USA	Maine	Sagadahoc	Richmond	ME
ME.2014.0137		2014-06-13	Gray Fox	USA	Maine	Cumberland	Naples	ME
ME.2014.0169		2014-06-26	Raccoon	USA	Maine	Washington	Deblois	ME
ME.2014.0197		2014-07-11	Raccoon	USA	Maine	Washington	Baileyville	ME
NB.2000.4394		2000-09-12	Skunk	Canada	New Brunswick	Charlotte	Heathland	NB
NB.2000.5443		2000-10-31	Skunk	Canada	New Brunswick	Charlotte	St. Stephen	NB
NB.2000.5579		2000-11-07	Raccoon	Canada	New Brunswick	Charlotte	St. Stephen	NB
NB.2000.5733		2000-11-16	Skunk	Canada	New Brunswick	Charlotte	St. Stephen	NB
NB.2000.5735		2000-11-16	Raccoon	Canada	New Brunswick	Charlotte	St. George	NB
NB.2001.0005		2001-01-02	Raccoon	Canada	New Brunswick	Charlotte	St. Stephen	NB
NB.2001.0006		2001-01-02	Skunk	Canada	New Brunswick	Charlotte	St. Stephen	NB
NB.2001.1588		2001-04-11	Raccoon	Canada	New Brunswick	Charlotte	Little Ridge	NB
NB.2001.3484		2001-04-26	Raccoon	Canada	New Brunswick	Charlotte	Dufferin	NB
NB.2001.6249		2001-06-21	Raccoon	Canada	New Brunswick	Charlotte	Cockerhill	NB
NB.2001.9134		2001-08-22	Raccoon	Canada	New Brunswick	Charlotte	St. Stephen	NB
NB.2002.3420		2002-05-30	Raccoon	Canada	New Brunswick	Charlotte	Valley Rd.	NB
NB.2014.0486		2014-06-02	Raccoon	Canada	New Brunswick	Charlotte	St. Stephen	NB
NB.2015.0046		2015-01-22	Raccoon	Canada	New Brunswick	Charlotte	Oak Bay	NB
NB.2015.0149		2015-03-03	Raccoon	Canada	New Brunswick	Charlotte	Waweig	NB
NB.2015.0155		2015-03-04	Raccoon	Canada	New Brunswick	Charlotte	Bayside	NB
NB.2015.0156		2015-03-04	Raccoon	Canada	New Brunswick	Charlotte	St. Stephen	NB
NB.2015.0183		2015-03-10	Raccoon	Canada	New Brunswick	Charlotte	Bocabec	NB
NB.2015.0185		2015-03-10	Raccoon	Canada	New Brunswick	Charlotte	Waweig	NB
NB.2015.0201		2015-03-18	Raccoon	Canada	New Brunswick	Charlotte	Burnt Hill	NB
NB.2015.0202		2015-03-18	Raccoon	Canada	New Brunswick	Charlotte	St. Stephen	NB
NB.2015.0207		2015-03-19	Raccoon	Canada	New Brunswick	Charlotte	Cooks Lane	NB
NB.2015.0270		2015-04-14	Raccoon	Canada	New Brunswick	Charlotte	Waweig	NB
NB.2015.0343		2015-05-05	Raccoon	Canada	New Brunswick	Charlotte	Waweig	NB
NB.2015.0359		2015-05-07	Raccoon	Canada	New Brunswick	Charlotte	Valley Rd.	NB
NB.2015.0583		2015-06-23	Raccoon	Canada	New Brunswick	Charlotte	St. Andrews	NB
NB.2015.0671		2015-07-14	Raccoon	Canada	New Brunswick	Charlotte	St. Andrews	NB
NB.2015.0672		2015-07-14	Raccoon	Canada	New Brunswick	Charlotte	Chamcook	NB
NB.2015.0774		2015-07-29	Skunk	Canada	New Brunswick	Charlotte	Bocabec	NB
NB.2015.0835		2015-08-06	Raccoon	Canada	New Brunswick	Charlotte	Bayside	NB
NB.2015.0921		2015-08-18	Raccoon	Canada	New Brunswick	Charlotte	Pennfield	NB
NB.2015.1049		2015-09-01	Raccoon	Canada	New Brunswick	York	McAdam	NB
NY.1994.1330		1994-03-24	Raccoon	USA	New York	Orleans	Kendall	NY west

Sequence ID	Accession no.	Date of testing	Host species	Country	Province/ State	County	Town	Geographic group
NY.1995.3745		1995-07-12	Raccoon	USA	New York	Clinton	Black Brook	VT
NY.1995.7951	KY026416	1995-12-22	Raccoon	USA	New York	Jefferson	Hounsfield	NY north-west
NY.1998.8982	KY026417	1998-11-09	Raccoon	USA	New York	St Lawrence	Canton	NY north-west
NY.1998.9581	KY026418	1998-12-08	Skunk	USA	New York	St Lawrence	Morristown	NY north-west
NY.2003.1694		2003-05-21	Raccoon	USA	New York	Cayuga	Sterling	NY north-west
NY.2003.1995		2003-06-06	Raccoon	USA	New York	Orleans	Kendall	NY west
NY.2003.7809		2003-11-24	Skunk	USA	New York	Orleans	Ridgeway	NY west
NY.2003.8109		2003-12-22	Raccoon	USA	New York	Niagara	Wheatfield	NY west
NY.2003.8176		2003-12-25	Raccoon	USA	New York	Orleans	Albion	NY west
NY.2004.0023		2004-01-05	Skunk	USA	New York	Oswego	Scriba	NY north-west
NY.2004.0353		2004-02-06	Raccoon	USA	New York	Wyoming	Middlebury	NY west
NY.2004.0953	KY026419	2004-04-06	Skunk	USA	New York	Jefferson	Brownville	NY north-west
NY.2004.1133		2004-04-21	Raccoon	USA	New York	Jefferson	Adams	NY north-west
NY.2004.1285		2004-05-04	Raccoon	USA	New York	Wayne	Williamson	NY north-west
NY.2004.1600		2004-05-20	Raccoon	USA	New York	Niagara	Niagara Falls	NY west
NY.2004.1974		2004-06-03	Raccoon	USA	New York	Cayuga	Sterling	NY north-west
NY.2004.2753		2004-06-30	Raccoon	USA	New York	Jefferson	Adams	NY north-west
NY.2004.4289		2004-08-04	Raccoon	USA	New York	Erie	Newstead	NY west
NY.2004.4875		2004-08-13	Raccoon	USA	New York	Orleans	Kendall	NY west
NY.2004.5301		2004-08-18	Raccoon	USA	New York	Wayne	Sodus	NY north-west
NY.2004.6938		2004-09-17	Skunk	USA	New York	Oswego	Oswego City	NY north-west
NY.2004.7916		2004-11-16	Skunk	USA	New York	Cayuga	Sterling	NY north-west
NY.2004.7972		2004-11-18	Skunk	USA	New York	Cayuga	Sterling	NY north-west
NY.2004.8025		2004-11-23	Raccoon	USA	New York	Jefferson	Watertown	NY north-west
NY.2010.1066		2010-04-08	Skunk	USA	New York	St Lawrence	Hammond	NY north-west
NY.2010.1178		2010-04-20	Raccoon	USA	New York	St Lawrence	Gouveneur	NY north-west
NY.2010.1233		2010-12-10	Raccoon	USA	New York	St Lawrence	Gouveneur	NY north-west
NY.2010.1234		2010-04-23	Skunk	USA	New York	Niagara	Cambria	NY west
NY.2010.1349		2010-05-04	Raccoon	USA	New York	Orleans	Yates	NY west
NY.2010.1444		2010-05-11	Raccoon	USA	New York	Chautauqua	Sherman	NY west
NY.2010.1962		2010-06-02	Raccoon	USA	New York	Chemung	Horseheads	NY west
NY.2010.1986		2010-06-02	Raccoon	USA	New York	Erie	Orchard Parl	NY west
NY.2010.2167		2010-06-09	Raccoon	USA	New York	Genesee	Byron	NY west
NY.2010.2369		2010-06-18	Skunk	USA	New York	Cayuga	Sterling	NY north-west
NY.2010.2456		2014-06-23	Raccoon	USA	New York	Niagara	Niagra	NY west
NY.2010.3956		2010-08-05	Skunk	USA	New York	Oswego	Oswego City	NY north-west
NY.2010.5558		2010-09-21	Raccoon	USA	New York	Niagara	Porter	NY west
NY.2010.5667		2010-09-29	Skunk	USA	New York	Chautauqua	Hanover	NY west
NY.2010.5742		2010-10-06	Raccoon	USA	New York	Orleans	Carlton	NY west
NY.2010.5965		2010-10-26	Skunk	USA	New York	Wyoming	Wethersfield	NY west
NY.2011.0862		2011-04-05	Raccoon	USA	New York	Jefferson	Adams	NY north-west
NY.2011.1089		2011-04-26	Raccoon	USA	New York	Erie	Amherst	NY west
NY.2011.1548	KY026421	2011-05-31	Raccoon	USA	New York	St Lawrence	De Kalb	NY north-west
NY.2011.2028		2011-06-16	Raccoon	USA	New York	Jefferson	Ellisburg	NY north-west
NY.2011.2880		2011-07-22	Cat	USA	New York	Wayne	Wolcott	NY north-west
NY.2011.4498		2011-08-19	Raccoon	USA	New York	Genesee	Batavia City	NY west
NY.2011.5028		2011-09-02	Raccoon	USA	New York	Erie	Amherst	NY west
NY.2011.5139		2011-09-08	Skunk	USA	New York	Jefferson	Water	NY north-west
NY.2011.5196		2011-09-13	Skunk	USA	New York	Lewis	Croghan	NY north-west
NY.2011.5335		2011-09-21	Skunk	USA	New York	Oswego	Mexico	NY north-west
NY.2011.5545		2011-10-06	Cat	USA	New York	Wayne	Ontario	NY north-west
NY.2011.5590		2011-10-07	Skunk	USA	New York	Clinton	Champlain	VT
NY.2011.5763		2011-10-21	Gray Fox	USA	New York	Oswego	Sandy Creek	NY north-west
ON.1999.3545	EU311738	1999-07-26	Raccoon	Canada	Ontario	Leeds	North Augusta	ON east
ON.1999.5025	KY026423	1999-09-17	Raccoon	Canada	Ontario	Grenville	Oxford Station	ON east
ON.1999.6417	KY026424	1999-12-10	Raccoon	Canada	Ontario	Frontenac	Wolfe Island	ON east
ON.1999.6479	KY026425	1999-12-16	Raccoon	Canada	Ontario	Leeds	North Augusta	ON east
ON.2000.0112	KY026426	2000-01-12	Raccoon	Canada	Ontario	Frontenac	Wolfe Island	ON east
ON.2000.0155	KY026427	2000-01-13	Raccoon	Canada	Ontario	Frontenac	Wolfe Island	ON east
ON.2000.0222	KY026428	2000-01-19	Raccoon	Canada	Ontario	Grenville	North Augusta	ON east
ON.2000.0484	KY026429	2000-02-14	Raccoon	Canada	Ontario	Grenville	Garretton	ON east
ON.2000.0771	KY026430	2000-03-07	Raccoon	Canada	Ontario	Leeds	North Augusta	ON east
ON.2000.1357	KY026432	2000-04-18	Raccoon	Canada	Ontario	Grenville	Bishops Mills	ON east
ON.2000.1730	KY026433	2000-05-17	Raccoon	Canada	Ontario	Grenville	Merrickville	ON east
ON.2000.1818	KY026434	2000-05-23	Raccoon	Canada	Ontario	Grenville	North Augusta	ON east
ON.2000.2067	KY026435	2000-06-05	Raccoon	Canada	Ontario	Leeds	New Dublin	ON east
ON.2000.2201	KY026436	2000-06-12	Raccoon	Canada	Ontario	Leeds	Addison	ON east

Sequence ID	Accession no.	Date of testing	Host species	Country	Province/ State	County	Town	Geographic group
ON.2000.2342	KY026437	2000-06-19	Raccoon	Canada	Ontario	Grenville	Bishops Mills	ON east
ON.2000.2398	KY026438	2000-06-20	Raccoon	Canada	Ontario	Grenville	Algonquin	ON east
ON.2000.4360	KY026439	2000-09-08	Raccoon	Canada	Ontario	Leeds	North Augusta	ON east
ON.2000.4461	KY026440	2000-09-15	Raccoon	Canada	Ontario	Leeds	Addison	ON east
ON.2000.4474	KY026441	2000-09-18	Raccoon	Canada	Ontario	Leeds	RR#5 Brockville	ON east
ON.2000.5320	KY026442	2000-10-25	Raccoon	Canada	Ontario	Leeds	Frankville	ON east
ON.2000.6152	KY026443	2000-12-14	Raccoon	Canada	Ontario	Leeds	Athens	ON east
ON.2001.0312	KY026444	2001-01-26	Raccoon	Canada	Ontario	Leeds	Black Church Rd.	ON east
ON.2001.0889	KY026445	2001-03-12	Raccoon	Canada	Ontario	Leeds	Graham Lake Rd.	ON east
ON.2001.1190	KY026446	2001-03-26	Raccoon	Canada	Ontario	Leeds	Upper Oak Leaf Rd.	ON east
ON.2001.12052	KY026447	2001-12-13	Raccoon	Canada	Ontario	Leeds	Bastard Twp	ON east
ON.2001.12053	KY026448	2001-12-13	Raccoon	Canada	Ontario	Leeds	Toledo	ON east
ON.2001.1891	KY026449	2001-04-18	Raccoon	Canada	Ontario	Leeds	Athens	ON east
ON.2001.1895	KY026450	2001-04-18	Skunk	Canada	Ontario	Leeds	Mallorytown	ON east
ON.2001.3744	KY026451	2001-04-30	Raccoon	Canada	Ontario	Leeds	Athens	ON east
ON.2001.3913	KY026452	2001-05-04	Raccoon	Canada	Ontario	Leeds	Charleston Village	ON east
ON.2001.4148	KY026453	2001-05-16	Raccoon	Canada	Ontario	Leeds	Rocksprings	ON east
ON.2001.6195	KY026454	2001-06-19	Raccoon	Canada	Ontario	Leeds	Athens	ON east
ON.2001.7006	KY026455	2001-07-03	Raccoon	Canada	Ontario	Leeds	Philipsville	ON east
ON.2001.7160	KY026456	2001-07-06	Raccoon	Canada	Ontario	Leeds	Athens	ON east
ON.2001.8399	KY026457	2001-08-08	Raccoon	Canada	Ontario	Leeds	Delta	ON east
ON.2001.9303	KY026458	2001-08-24	Raccoon	Canada	Ontario	Leeds	Brockville	ON east
ON.2002.3398	KY026459	2002-05-29	Raccoon	Canada	Ontario	Leeds	Kitley Twp	ON east
ON.2002.3853	KY026460	2002-06-21	Raccoon	Canada	Ontario	Leeds	Elgin	ON east
ON.2002.7095	KY026461	2002-10-02	Raccoon	Canada	Ontario	Grenville	Ventnor Rd.	ON east
ON.2002.7849	KY026462	2002-10-24	Raccoon	Canada	Ontario	Grenville	Ventnor Rd.	ON east
ON.2002.8206	KY026463	2002-11-05	Raccoon	Canada	Ontario	Leeds	Mallorytown	ON east
ON.2003.0941	KY026464	2003-02-21	Raccoon	Canada	Ontario	Grenville	Spencerville	ON east
ON.2003.1519	KY026465	2003-03-19	Raccoon	Canada	Ontario	Grenville	Prescott	ON east
ON.2003.2673	KY026466	2003-04-14	Raccoon	Canada	Ontario	Grenville	Spencerville	ON east
ON.2003.2760	KY026467	2003-04-17	Raccoon	Canada	Ontario	Leeds	Athens	ON east
ON.2003.2794	KY026468	2003-04-22	Raccoon	Canada	Ontario	Leeds	Spencerville	ON east
ON.2003.3406	KY026469	2003-05-05	Raccoon	Canada	Ontario	Leeds	Cardinal	ON east
ON.2003.3574	KY026470	2003-05-12	Raccoon	Canada	Ontario	Leeds	Kemptville	ON east
ON.2003.4680	KY026471	2003-06-10	Raccoon	Canada	Ontario	Grenville	Prescott	ON east
ON.2003.5257	KY026472	2003-06-30	Raccoon	Canada	Ontario	Leeds	Mallorytown	ON east
ON.2004.6275	KY026473	2004-08-13	Raccoon	Canada	Ontario	Leeds	Mallorytown	ON east
ON.2004.6277	KY026474	2004-08-13	Raccoon	Canada	Ontario	Leeds	Mallorytown	ON east
ON.2004.6953	KY026475	2004-08-31	Raccoon	Canada	Ontario	Leeds	Mallorytown	ON east
ON.2004.7702	KY026476	2004-09-22	Raccoon	Canada	Ontario	Leeds	Mallorytown	ON east
ON.2005.4941	KY026477	2005-09-22	Raccoon	Canada	Ontario	Leeds	Mallorytown	ON east
ON.2015.1361		2015-12-04	Raccoon	Canada	Ontario	Hamilton	Hamilton	ON west
QC.2006.2049		2006-06-02	Raccoon	Canada	Quebec	Brome-Missisquoi	Dunham	QC
QC.2006.4930		2006-09-06	Raccoon	Canada	Quebec	Brome-Missisquoi	Cowansville	QC
QC.2006.5982		2006-11-15	Raccoon	Canada	Quebec	Brome-Missisquoi	Dunham	QC
QC.2007.0909		2007-08-01	Raccoon	Canada	Quebec	Brome-Missisquoi	Mont-Saint-Gregoire	QC
QC.2007.0910		2007-08-02	Raccoon	Canada	Quebec	Brome-Missisquoi	Mont-Saint-Gregoire	QC
QC.2007.1065		2007-08-29	Raccoon	Canada	Quebec	Brome-Missisquoi	Saint-Jean-sur-Richelieu	QC
QC.2007.1122		2007-08-30	Raccoon	Canada	Quebec	Brome-Missisquoi	Saint-Jean-sur-Richelieu	QC
QC.2007.2234		2007-05-11	Raccoon	Canada	Quebec	Brome-Missisquoi	Saint-Armand	QC
QC.2007.2783		2007-06-11	Raccoon	Canada	Quebec	Brome-Missisquoi	Frelighsburg	QC
QC.2007.2892		2007-06-13	Raccoon	Canada	Quebec	Brome-Missisquoi	Saint-Armand	QC
QC.2007.2893		2007-06-13	Raccoon	Canada	Quebec	Brome-Missisquoi	Frelighsburg	QC
QC.2007.2937		2007-06-13	Raccoon	Canada	Quebec	Brome-Missisquoi	Saint-Armand	QC

Sequence ID	Accession no.	Date of testing	Host species	Country	Province/ State	County	Town	Geographic group
QC.2007.2939		2007-06-13	Raccoon	Canada	Quebec	Brome-Missisquoi	Saint-Pierre-de-Veronne-a-Pike-River	QC
QC.2007.2941		2007-06-13	Raccoon	Canada	Quebec	Brome-Missisquoi	Stanbridge East	QC
QC.2007.2942		2007-06-13	Raccoon	Canada	Quebec	Brome-Missisquoi	Saint-Armand	QC
QC.2007.2975		2007-06-14	Raccoon	Canada	Quebec	Brome-Missisquoi	Saint-Armand	QC
QC.2007.2986		2007-06-16	Raccoon	Canada	Quebec	Brome-Missisquoi	Saint-Pierre-de-Veronne-a-Pike-River	QC
QC.2007.3122		2007-06-21	Raccoon	Canada	Quebec	Brome-Missisquoi	Saint-Pierre-de-Veronne-a-Pike-River	QC
QC.2007.3211		2007-06-29	Raccoon	Canada	Quebec	Brome-Missisquoi	Saint-Sebastien	QC
QC.2007.3246		2007-07-03	Raccoon	Canada	Quebec	Brome-Missisquoi	Saint-Sebastien	QC
QC.2007.3657		2007-07-05	Raccoon	Canada	Quebec	Brome-Missisquoi	Frelighsburg	QC
QC.2007.3658		2007-07-14	Raccoon	Canada	Quebec	Brome-Missisquoi	Noyan	QC
QC.2007.3659		2007-07-15	Raccoon	Canada	Quebec	Brome-Missisquoi	Noyan	QC
QC.2007.3870		2007-07-19	Raccoon	Canada	Quebec	Brome-Missisquoi	Saint-Pierre-de-Veronne-a-Pike-River	QC
QC.2007.4485		2007-08-03	Raccoon	Canada	Quebec	Brome-Missisquoi	Saint-Armand	QC
QC.2007.5318		2007-08-23	Raccoon	Canada	Quebec	Brome-Missisquoi	Bedford	QC
QC.2007.5827		2007-09-21	Raccoon	Canada	Quebec	Le Haut-Richelieu	Sainte-Anne-de-Sabrevois	QC
QC.2007.6184		2007-10-11	Raccoon	Canada	Quebec	Brome-Missisquoi	Stanbridge Station	QC
QC.2007.6234		2007-10-12	Skunk	Canada	Quebec	Brome-Missisquoi	Cowansville	QC
QC.2007.6292		2007-11-01	Raccoon	Canada	Quebec	Le Haut-Richelieu	Saint-Jean-sur-Richelieu	QC
QC.2007.6389		2007-11-01	Skunk	Canada	Quebec	Le Haut-Richelieu	Saint-Alexandre	QC
QC.2007.6390		2007-10-20	Raccoon	Canada	Quebec	Le Haut-Richelieu	Noyan	QC
QC.2007.6442		2007-11-15	Red Fox	Canada	Quebec	Le Haut-Richelieu	Saint-Jean-sur-Richelieu	QC
QC.2007.6659		2007-11-19	Raccoon	Canada	Quebec	Le Haut-Richelieu	Noyan	QC
QC.2008.0750		2008-03-04	Raccoon	Canada	Quebec	Brome-Missisquoi	Farnham	QC
QC.2008.1096		2008-03-17	Raccoon	Canada	Quebec	Brome-Missisquoi	Farnham	QC
QC.2008.1237		2008-03-21	Raccoon	Canada	Quebec	Le Haut-Richelieu	Noyan	QC
QC.2008.1411		2008-04-15	Raccoon	Canada	Quebec	Brome-Missisquoi	Sutton	QC
QC.2008.1846		2008-05-07	Raccoon	Canada	Quebec	Le Haut-Richelieu	Noyan	QC
QC.2008.1848		2008-05-08	Raccoon	Canada	Quebec	Le Haut-Richelieu	Farnham	QC
QC.2008.1894		2008-05-13	Raccoon	Canada	Quebec	Brome-Missisquoi	Farnham	QC
QC.2008.1970		2008-05-13	Raccoon	Canada	Quebec	Le Haut-Richelieu	Noyan	QC
QC.2008.2444		2008-06-16	Raccoon	Canada	Quebec	Le Haut-Richelieu	Saint-Georges-de-Clarenceville	QC
QC.2008.4180		2008-07-31	Skunk	Canada	Quebec	Le Haut-Richelieu	Sainte-Anne-de-Sabrevois	QC
QC.2008.4262		2008-08-25	Raccoon	Canada	Quebec	Brome-Missisquoi	Sainte-Sabine	QC
QC.2008.4808		2008-09-03	Raccoon	Canada	Quebec	Le Haut-Richelieu	Henryville	QC

Sequence ID	Accession no.	Date of testing	Host species	Country	Province/ State	County	Town	Geographic group
QC.2008.5000		2008-09-17	Raccoon	Canada	Quebec	Rouville	Ange-Gardien	QC
QC.2008.5001		2008-09-20	Raccoon	Canada	Quebec	Rouville	Saint-Cesaire	QC
QC.2008.5337		2008-10-15	Skunk	Canada	Quebec	Le Haut-Richelieu	Henryville	QC
QC.2009.0709		2009-02-26	Skunk	Canada	Quebec	Le Haut-Richelieu	Clarenceville	QC
QC.2009.0819		2009-04-07	Skunk	Canada	Quebec	Le Haut-Richelieu	Clarenceville	QC
QC.2015.0488		2015-06-04	Raccoon	Canada	Quebec	Akwesasne Reservation	St Regis	ON east
VT.2005.0215		2005-12-29	Raccoon	USA	Vermont	Washington	Cabot	VT
VT.2006.0104		2006-08-29	Raccoon	USA	Vermont	Caledonia	St. Johnsbury	VT
VT.2006.0108		2006-08-30	Raccoon	USA	Vermont	Lamoille	Stowe	VT
VT.2006.0116		2006-09-05	Cow	USA	Vermont	Caledonia	Danville	VT
VT.2006.0117		2006-09-05	Skunk	USA	Vermont	Chittenden	South Burlington	VT
VT.2006.0153		2006-10-04	Skunk	USA	Vermont	Lamoille	Stowe	VT
VT.2006.0166		2006-10-11	Raccoon	USA	Vermont	Rutland	Clarendon	VT
VT.2006.0178		2006-10-30	Skunk	USA	Vermont	Addison	Panton	VT
VT.2006.0225	KY026478	2006-11-17	Raccoon	USA	Vermont	Lamoille	Stowe	VT
VT.2006.0332		2006-05-23	Skunk	USA	Vermont	Addison	Middlebury	VT
VT.2007.0084		2007-07-20	Skunk	USA	Vermont	Chittenden	Burlington	VT
VT.2007.0092		2007-07-25	Raccoon	USA	Vermont	Caledonia	Lyndon	VT
VT.2007.0297		2007-01-04	Raccoon	USA	Vermont	Lamoille	Stowe	VT
VT.2007.0308		2007-01-31	Raccoon	USA	Vermont	Bennington	Pownal	VT
VT.2007.0320		2007-09-11	Skunk	USA	Vermont	Chittenden	Williston	VT
VT.2007.0342		2007-03-02	Raccoon	USA	Vermont	Franklin	St. Albans City	VT
VT.2007.0380		2007-09-18	Raccoon	USA	Vermont	Franklin	Fairfield	VT
VT.2007.0392		2007-03-22	Raccoon	USA	Vermont	Chittenden	Burlington	VT
VT.2007.0398		2007-09-26	Skunk	USA	Vermont	Franklin	Georgia	VT
VT.2007.0412		2007-03-30	Raccoon	USA	Vermont	Chittenden	Essex	VT
VT.2007.0465		2007-10-15	Skunk	USA	Vermont	Franklin	Sheldon	VT
VT.2007.0479		2007-10-30	Skunk	USA	Vermont	Franklin	Swanton	VT
VT.2007.0489		2007-11-05	Raccoon	USA	Vermont	Grand Isle	North Hero	VT
VT.2007.0491		2007-11-05	Skunk	USA	Vermont	Franklin	Franklin	VT
VT.2007.0492		2007-04-25	Raccoon	USA	Vermont	Caledonia	Danville	VT
VT.2007.0496		2007-04-25	Raccoon	USA	Vermont	Caledonia	Danville	VT
VT.2007.0505		2007-04-30	Skunk	USA	Vermont	Caledonia	Danville	VT
VT.2007.0506		2007-11-14	Skunk	USA	Vermont	Franklin	Georgia	VT
VT.2007.0512		2007-11-20	Skunk	USA	Vermont	Franklin	Sheldon	VT
VT.2007.0523		2007-12-10	Skunk	USA	Vermont	Franklin	Franklin	VT
VT.2008.0237	KY026482	2008-09-29	Raccoon	USA	Vermont	Lamoille	Stowe	VT
VT.2008.0270		2008-11-06	Skunk	USA	Vermont	Lamoille	Elmore	VT
VT.2008.0310		2008-12-29	Cow	USA	Vermont	Franklin	Bakersfield	VT
VT.2009.0036		2009-07-20	Skunk	USA	Vermont	Franklin	Fairfax	VT
VT.2009.0092		2009-08-14	Raccoon	USA	Vermont	Grand Isle	South Hero	VT
VT.2009.0093		2009-08-20	Skunk	USA	Vermont	Orleans	Craftsbury	VT
VT.2009.0124		2009-10-04	Skunk	USA	Vermont	Chittenden	Williston	VT
VT.2009.0134		2009-10-12	Skunk	USA	Vermont	Caledonia	Sheffield	VT
VT.2009.0136		2009-10-19	Raccoon	USA	Vermont	Chittenden	Essex	VT
VT.2009.0146		2009-10-19	Raccoon	USA	Vermont	Orleans	Craftsbury	VT
VT.2009.0164		2009-11-05	Raccoon	USA	Vermont	Franklin	Franklin	VT
VT.2009.0169		2009-11-01	Skunk	USA	Vermont	Chittenden	Shelburne	VT
VT.2009.0194		2009-11-30	Raccoon	USA	Vermont	Chittenden	Williston	VT
VT.2009.0352		2009-02-26	Raccoon	USA	Vermont	Grand Isle	Grand Isle	VT
VT.2009.0370		2009-03-17	Raccoon	USA	Vermont	Lamoille	Stowe	VT
VT.2009.0371		2009-03-11	Cow	USA	Vermont	Orleans	Barton	VT
VT.2009.0397		2009-04-05	Cow	USA	Vermont	Orleans	Greensboro	VT
VT.2009.0423		2009-04-17	Raccoon	USA	Vermont	Orleans	Greensboro	VT
VT.2009.0428		2009-04-17	Raccoon	USA	Vermont	Franklin	Franklin	VT
VT.2009.0443		2009-05-12	Cow	USA	Vermont	Lamoille	Hyde Park	VT
VT.2010.0044		2010-07-29	Skunk	USA	Vermont	Chittenden	Burlington	VT
VT.2010.0054		2010-08-09	Raccoon	USA	Vermont	Chittenden	South Burlington	VT
VT.2010.0089		2010-08-20	Raccoon	USA	Vermont	Chittenden	Burlington	VT
VT.2010.0090		2010-08-23	Skunk	USA	Vermont	Chittenden	Winooski	VT
VT.2010.0093		2010-08-29	Skunk	USA	Vermont	Chittenden	Winooski	VT
VT.2010.0145		2010-10-14	Skunk	USA	Vermont	Orleans	Craftsbury	VT

Sequence ID	Accession no.	Date of testing	Host species	Country	Province/ State	County	Town	Geographic group
VT.2010.0172		2010-11-18	Skunk	USA	Vermont	Chittenden	South Burlington	VT
VT.2010.0370		2010-06-18	Skunk	USA	Vermont	Chittenden	Burlington	VT
VT.2011.0079		2011-08-27	Skunk	USA	Vermont	Chittenden	Burlington	VT
VT.2011.0122	KY026483	2011-10-23	Skunk	USA	Vermont	Caledonia	Walden	VT
VT.2011.0138		2011-11-04	Skunk	USA	Vermont	Orleans	Derby	VT
VT.2011.0198		2011-01-08	Raccoon	USA	Vermont	Chittenden	Burlington	VT

Technical Appendix Table 2. Summary of phylogenetic model selection path sampling (PS) and stepping stone sampling (SS) results.

Nt. subs. model	Gene partitions	Codon partitions	UCLD clock model		UCED clock model	
			PS	SS	PS	SS
GTR+G	5x genes + non-coding	1a) None	-41504	-40505	-39250	-38101
		1b) 3 partitions	*	*	-37408	-35381
	Coding regions + non-coding	2a) None	-41912	-40675	-18312	-18312
		2b) 3 partitions	-42270	-42274	-42278	-42283
	None	3) None	-43239	-43244	-43260	-43266

*UCLD model 1b) failed to converge.

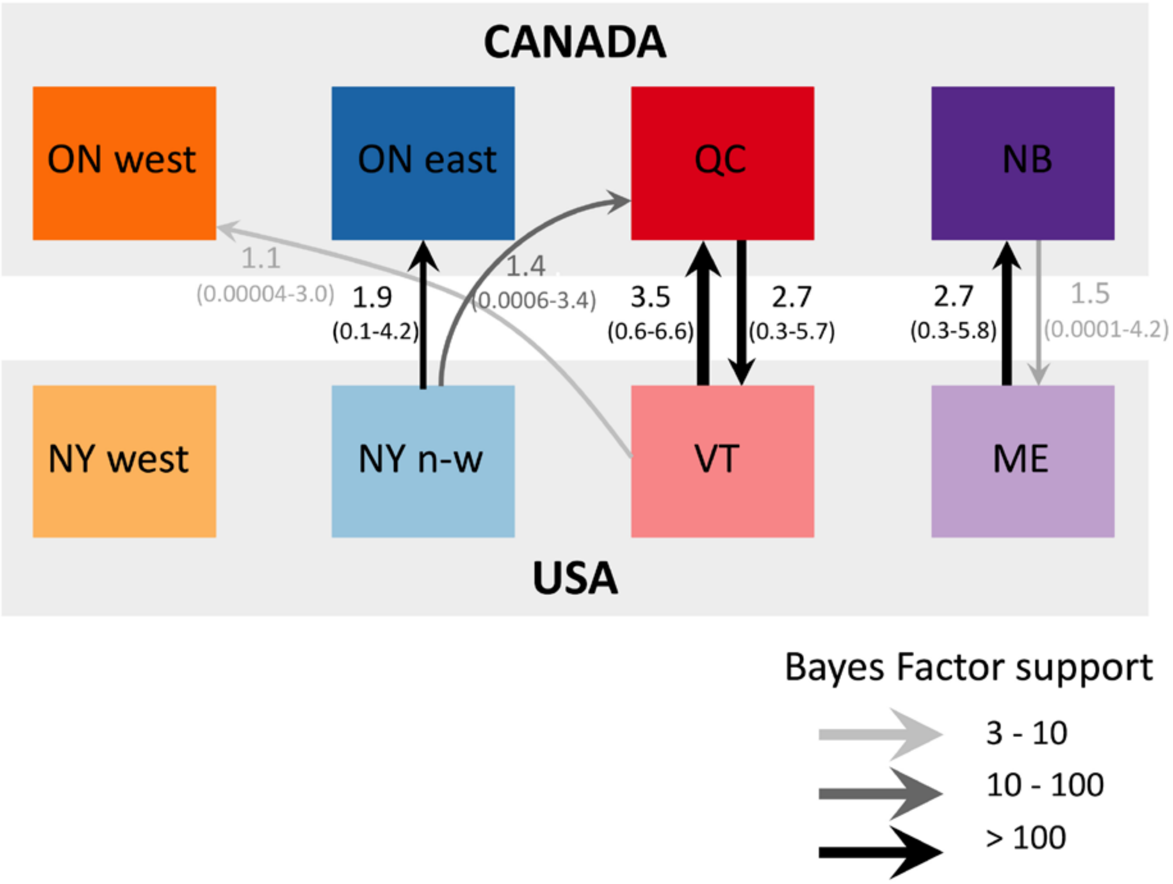

Technical Appendix Figure. RRV transitions between different regions of Canada and USA, estimated using discrete trait phylogeography. Arrows are shaded according to statistical support for the transition. The estimated number of transitions between regions is given next to the relevant arrow, with the 95% Highest Posterior Density in brackets.
